# Adult Stem Cells in Tissue Maintenance and Regeneration

**DOI:** 10.1155/2016/7362879

**Published:** 2016-02-02

**Authors:** Stefania Montagnani, Maria A. Rueger, Toru Hosoda, Daria Nurzynska

**Affiliations:** ^1^Department of Public Health, University of Naples “Federico II”, 80131 Naples, Italy; ^2^Department of Neurology, University Hospital of Cologne, 50924 Cologne, Germany; ^3^Medical Science Division, Tokai University Institute of Innovative Science and Technology, Kanagawa 259-1193, Japan

Adult stem cells, also known as somatic stem cells, reside in specific tissues and possess fundamental properties of stem cells, that is, self-renewal capacity and, though limited in magnitude, multipotency. Generally, these primitive cells are stored in a specialized environment called niche, where they are connected to supporting cells, protected from external harmful stimuli, and kept quiescent until the arrival of an appropriate activating signal. In accordance with the demand of the organ, tissue-specific adult stem cells proliferate, migrate to leave the niche, and differentiate to replace senescent or deteriorated cells, maintaining the organ structure and function. Also, they are known to repair mild injuries in various organs including the skin, liver, intestine, kidney, and bone marrow. Such endogenous regenerative mechanisms, however, appear insufficient to cope with severe damage, as in the case of myocardial infarction [1, 2] or cerebral ischemia [3], in which the damage is mostly irreversible despite the presence of local stem cells. Possibly, tissue-specific biological cues that determine the fate of adult stem cells and their committed progenitors in normal and pathological conditions pose limits to cell differentiation and survival* in vivo*.

While our knowledge on adult stem cells is expanding, the abundance and ever growing number of original research articles, which often raise new questions rather than yield conclusive answers, are a call for the need to summarize information and gather the perspective necessary to focus the interests and direct future scientific efforts toward the clinical application. Such was the purpose of this special issue, composed of both the original research papers, dealing with current stem cell therapy approaches, and the concise review articles, summarizing the current notion of tissue regeneration and reporting up-to-date knowledge on topics related to adult tissue-specific stem cells.

The special issue opens with a review by A. Klimczak and U. Kozlowska, which gives an overview of the current knowledge of biology of adult mesenchymal stem and progenitor cells in organs such as bone marrow, liver, skeletal muscle, skin, heart, and lung. Importantly, the authors stress that both stem cell differentiation capabilities and paracrine influence on other tissue-resident cells could be considered as a trigger for organ regeneration and function maintenance. The following reviews are dedicated each to a specific organ or tissue, namely, liver, heart, dental pulp, and skin.

First, G. Carpino et al. describe the hierarchical structure of the adult stem cells and their respective niches in the liver; there are at least three distinct niches, although their relationship is yet to be demonstrated. In this review, the possible role of stem/progenitor cells in the physiological cell turnover, liver, and biliary disease progression and regenerative activity and the potential strategy for cell therapy are discussed.

Second, M. I. Schaun and colleagues summarize the current experience with myocardial regeneration by stem cell transplantation. Interestingly, the authors argue that both pharmacological and nonpharmacological approaches to cell and their microenvironment modification should be taken in consideration when attempting to enhance cell survival, migration, and maturation.

Next, E. Ledesma-Martínez et al. shed light on dental pulp stem cells. These cells, derived from deciduous teeth, can generate structures such as dentin, periodontal ligament, and dental pulp, but they can also be considered a cell source for orthopaedic and maxillofacial reconstruction. The authors describe the state-of-the-art in isolation, characterization, and use of this cell population in tissue regeneration in preclinical and clinical setting.

In the final review of the series, D. Chen et al. explore the role of adult mesenchymal stem cells in wound healing, focusing in particular on their contribution to reepithelialization. While undoubtedly the paracrine angiogenic and anti-inflammatory action of stromal cells can be a determinant of proper wound healing, there is also evidence that these cells can differentiate into keratinocytes. The authors mention also yet another approach to adult organ regeneration, namely, tissue engineering, with the use of skin-compatible biomaterials seeded with mesenchymal stem cells.

The original research papers included in the present special issue are relevant to the current problems that science and medicine still cope with in the attempt to induce tissue regeneration. Unsurprisingly, the availability and ease of isolation of cells are determined by their source; hence, the mesenchymal stem cells of blood, bone marrow, and adipose tissue origin are the most studied populations of adult stem cells. In this regard, N. Zhu and colleagues investigate human mesenchymal stem cells resident in the bone marrow; in their original research article, they depicted the pivotal role of a nuclear receptor NR2F2 in the stemness of these cells. Next, V. Angelou et al. present evidence that autologous mesenchymal stem cells derived from adipose tissue facilitate regeneration in chronic scar lesions to the vocal folds. Last, R. Stojko et al. compare stem cells derived from umbilical cord blood and bone marrow. They report differences in the gene expression of the components of a number of signaling pathways, providing a reference database for successive researchers.

In summary, our understanding of adult tissue-specific stem cell biology can provide the basis for experimental and therapeutic tissue regeneration. Moving on to a clinical setting, the application of tissue-specific adult stem cells is in many regards more acceptable and safer than embryonic/fetal or induced pluripotent stem cells and thereby closer to the medical application. Still, only established and verified protocols for cell isolation, expansion, modification, and transplantation should be introduced in the clinic together with the ethical and legal norms regulating the use of human tissue.

*Stefania Montagnani* *Stefania Montagnani* *Maria A. Rueger* *Maria A. Rueger* *Toru Hosoda* *Toru Hosoda* *Daria Nurzynska* *Daria Nurzynska*

## References

[1] Hosoda T., Rota M., Kajstura J., Leri A., Anversa P. (2011). Role of stem cells in cardiovascular biology. *Journal of Thrombosis and Haemostasis*.

[2] Nurzynska D., Di Meglio F., Romano V. (2013). Cardiac primitive cells become committed to a cardiac fate in adult human heart with chronic ischemic disease but fail to acquire mature phenotype: genetic and phenotypic study. *Basic Research in Cardiology*.

[3] Rueger M. A., Schroeter M. (2015). In vivo imaging of endogenous neural stem cells in the adult brain. *World Journal of Stem Cells*.

